# Silver and Copper Acute Effects on Membrane Proteins and Impact on Photosynthetic and Respiratory Complexes in Bacteria

**DOI:** 10.1128/mBio.01535-18

**Published:** 2018-11-20

**Authors:** Reem Tambosi, Sylviane Liotenberg, Marie-Line Bourbon, Anne-Soisig Steunou, Marion Babot, Anne Durand, Nouari Kebaili, Soufian Ouchane

**Affiliations:** a Institute for Integrative Biology of the Cell (I2BC), Université Paris Saclay, CEA, CNRS, Université Paris Sud, Gif-sur-Yvette, France; b Laboratoire Aimé Cotton (LAC), CNRS, Université Paris Sud, ENS Paris Saclay, Campus d’Orsay, Université Paris Sud, Orsay, France; University of Pennsylvania; University of Chicago

**Keywords:** chlorophyll, copper, membrane complexes, metal homeostasis, photosynthesis, respiration, silver, toxicity

## Abstract

Silver (Ag^+^) and copper (Cu^+^) ions have been used for centuries in industry, as well as antimicrobial agents in agriculture and health care. Nowadays, Ag^+^ is also widely used in the field of nanotechnology. Yet, the underlying mechanisms driving toxicity of Ag^+^ ions *in vivo* are poorly characterized. It is well known that exposure to excess metal impairs the photosynthetic apparatus of plants and algae. Here, we show that the light-harvesting complex II (LH2) is the primary target of Ag^+^ and Cu^+^ exposure in the purple bacterium Rubrivivax gelatinosus. Ag^+^ and Cu^+^ specifically inactivate the 800-nm absorbing bacteriochlorophyll *a* (B800), while Ni^2+^ or Cd^2+^ treatment had no effect. This was further supported by analyses of CuSO_4_- or AgNO_3_-treated membrane proteins. Indeed, this treatment induced changes in the LH2 absorption spectrum related to the disruption of the interaction of B800 molecules with the LH2 protein. This caused the release of B800 molecules and subsequently impacted the spectral properties of the carotenoids within the 850-nm absorbing LH2. Moreover, previous studies have suggested that Ag^+^ can affect the respiratory chain in mitochondria and bacteria. Our data demonstrated that exposure to Ag^+^, both *in vivo* and *in vitro*, caused a decrease of cytochrome *c* oxidase and succinate dehydrogenase activities. Ag^+^ inhibition of these respiratory complexes was also observed in *Escherichia coli*, but not in Bacillus subtilis.

## Abstract (precis)

The use of metal ions represents a serious threat to the environment and to all living organisms because of the acute toxicity of these ions. Nowadays, silver nanoparticles are one of the most widely used nanoparticles in various industrial and health applications. The antimicrobial effect of nanoparticles is in part related to the released Ag^+^ ions and their ability to interact with bacterial membranes. Here, we identify, both *in vitro* and *in vivo*, specific targets of Ag^+^ ions within the membrane of bacteria. This include complexes involved in photosynthesis, but also complexes involved in respiration.

## INTRODUCTION

Metal accumulation in the environment results in toxicity and defects in metabolism, leading to impaired growth of microorganisms, as well as to a variety of metabolic disorders in higher organisms. In most bacteria, metals such as Cu^+^, Cd^2+^, or Ag^+^ would diffuse through nonspecific importers within the membrane. This induces the expression of the detoxification systems that allow the cell to tolerate the presence of metals in its environment (1–6). Among these systems, metal efflux systems are very efficient to detoxify excess metal. The P_1_B-type ATPases are the most frequently present heavy metal transporters in bacteria (7). They extrude excess or toxic metal ions such as Cu^+^, Zn^2+^, Cd^2+^, Co^2+^, Pb^2+^, or Ag^+^ from the cytoplasm to the periplasm, where metal is handled by other detoxifying proteins. In Escherichia coli, the Cu^+^ detoxifying system includes the Cu^+^ efflux ATPase CopA, the CusFCBA efflux system, and the CueO oxidase (8, 9). These systems are also involved in Ag^+^ detoxification in E. coli and other species (9–11). In mutants defective in the efflux system, metal accumulation in the cytoplasm can disrupt different metabolic pathways. Indeed, Cu^+^, Ag^+^, or Cd^2+^ can disrupt the solvent-exposed 4Fe-4S clusters of dehydratases (12, 13). In the purple photosynthetic bacterium Rubrivivax gelatinosus, Cu^+^ induces the expression of the CopA-ATPase and the periplasmic blue copper protein CopI (14, 15). Recent *in vivo* studies showed that Cu^+^ accumulation in R. gelatinosus and the human pathogen Neisseria gonorrhoeae Δ*copA* mutants affects cell growth by altering heme biosynthesis in the cytoplasm (14, 16) or cytochrome *c* assembly in the periplasm for the Δ*copI* mutant in R. gelatinosus (15). Interestingly, similar effect of tellurite on cytochrome *c-*type assembly was recently reported in Rhodobacter capsulatus (17). Cu^+^ can also compete with iron for the metal binding site of IscA and inhibit the 4Fe-4S cluster assembly pathway in E. coli (18). In plants and algae, metals exert their toxic action mostly by damaging chloroplasts, which leads to decreased efficiency of photosynthesis. Plants subject to excess metals usually exhibit a decrease in the photosystem amount and chlorophyll content (19–22). However, the toxicity mechanisms are not well known. Assessing the effect of metals on the growth of photosynthetic bacteria can provide new insights into the toxicity mechanisms and identify metal targets in phototrophs. Purple photosynthetic nonsulfur bacteria can grow by aerobic and anaerobic respiration or photosynthetically in the light under anaerobic or microaerobic conditions, using a cyclic electron transport chain. Aerobic respiration usually involves a branched energy-transducing electron transfer chain (23). The cytochrome *c*-dependent branch usually involves the NADH dehydrogenase, succinate dehydrogenase, the *bc_1_* complex, and the terminal cytochrome *c* oxidase (*aa*_3_ or *cbb*_3_). Under light-exposed condition, photosynthesis takes place within the membranous photosynthetic apparatus. The photosystem is usually composed of three pigment-protein complexes, namely, the two light-harvesting antennae (light-harvesting complex I [LH1] and light-harvesting complex II [LH2]) and the reaction center (RC), associated with carotenoids and bacteriochlorophylls (24). During the process, the light-harvesting complexes (LH) capture light energy and direct it to the RC, where conversion of the excitation energy/charge separation takes place. The LH antenna system consists of two large pigment-protein complexes, the core light-harvesting complex, LH1, that surrounds the RC, and the peripheral light-harvesting complex, LH2, induced under low-light conditions to increase light trapping efficiency in some species. Both LH antennae are composed of two integral membrane polypeptides (α and β) that associate with bacteriochlorophyll (BChl) and carotenoid molecules (25–27). The LH2 antennae contain two spectrally distinct bacteriochlorophylls, *a* (B800) and B850, which absorb in the near-infrared range, at 800 and 850 nm, respectively. The crystal structure of the LH2 from Rhodopseudomonas acidophila was previously resolved (25). The B850 molecules are sandwiched between the α and β subunits and are perpendicular to the membrane surface. In contrast, the B800 molecules are localized between the β subunits and aligned parallel to the membrane surface. The structures of the RC-LH1 core complexes of Rhodopseudomonas palustris and Thermochromatium tepidum are available (26, 27). In this study, we analyzed the effect of extended exposure to metals on photosynthesis and respiration in the photosynthetic purple bacterium R. gelatinosus. The data indicated that the B800 of LH2 was specifically removed upon exposure to AgNO_3_ and CuSO_4_. We then assessed the impact on the respiratory chain and showed that metal ions also damaged the succinate dehydrogenase and the terminal cytochrome *c* oxidase, thereby affecting respiration.

## RESULTS

### Silver is highly toxic for R. gelatinosus, and the Cu^+^-ATPase CopA is not involved in Ag^+^ response.

**FIG 1:**
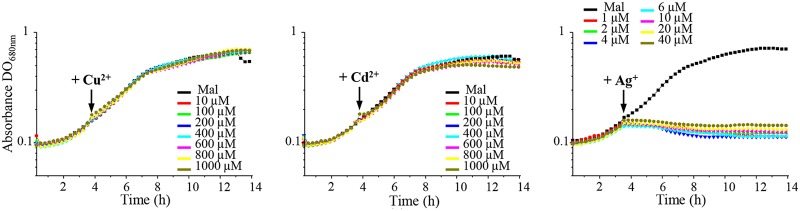
AgNO_3_ growth inhibition of R. gelatinosus. Wild-type cells were grown in 96-well microplates in the Tecan Infinite M200 luminometer. Indicated concentrations of CuSO_4_, CdCl_2_, or AgNO_3_ were added to the growth medium after 3.5 h, when cells reached an OD_680_ of 0.16 (arrow). Black line represents growth curves of untreated cells (malate) and red, green, blue, cyan, magenta, yellow, and brown lines represent growth curves of cells treated with increasing concentrations of Cu^2+^, Cd^2+^, or Ag^+^, as indicated.

**FIG 2:**
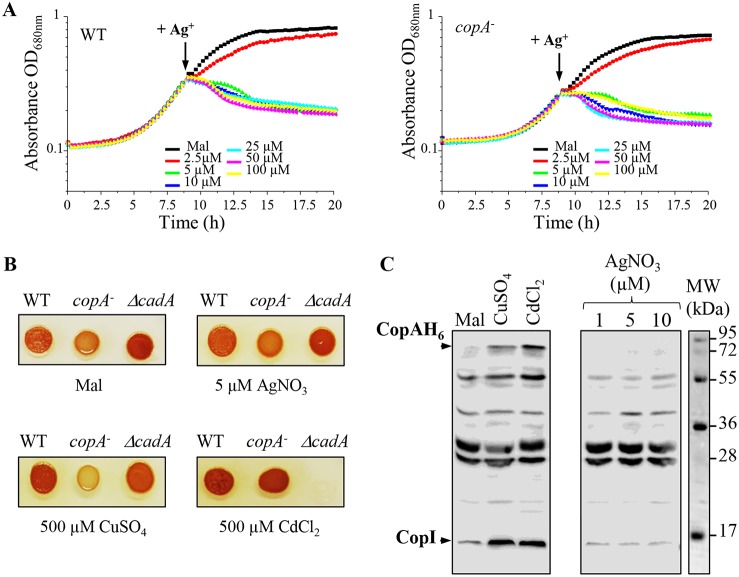
CopA and CadA are not involved in AgNO_3_ response. (A) Wild-type (WT) and *copA* mutant (*copA*^−^) cells were grown in microplates under microaerobic conditions. Indicated concentrations of AgNO_3_ were added to the growth medium after 8.5 h, when cells reached an OD_680_ of 0.3 (arrow). (B) Growth phenotype of the WT, *copA* and Δ*cadA* mutants in the presence of indicated concentrations of AgNO_3_, CuSO_4_, or CdCl_2_ on solid malate media. Cells were grown aerobically for 24 h at 30°C prior to photography. (C) Induction of CopA-H_6_ and CopI expression in cells shocked for 1 h with 0.5 mM CuSO_4_, 0.5 mM CdCl_2_, or 1 to 10 µM AgNO_3_. Cells were grown in photosynthetic condition and metals were added to the growth medium when cells reached an OD_680_ of 0.8. Total protein extracts from the same amount of cells (OD_680_ of 0.1) were separated on 14% SDS-PAGE. The proteins were revealed on a Western blot using an HisProbe-HRP.

To assess the toxicity of Ag^+^ in comparison with those of other toxic metals, wild-type cells were treated with increasing concentration of AgNO_3_, CuSO_4_, or CdCl_2_ during the exponential growth phase, and overnight growth was monitored. Growth was not affected by the addition of CuSO_4_ or CdCl_2_, even at 1 mM. In contrast, addition of 1 µM AgNO_3_ was enough to fully inhibit growth (Fig. 1). Similar results were reported in E. coli cells, highlighting the acute toxicity of Ag^+^ compared to that of other metal cations (13). We should note that toxicity was reduced when AgNO_3_ was added to a higher density of cells (Fig. S1), as previously reported for E. coli. It was suggested that metal ions could interact and be sequestered on the cell surface; the high cell density will therefore affect the dose response (13). To cope with excess toxic metal, bacteria usually induce the genes encoding the metal-efflux ATPases. Ag^+^ tolerance in E. coli involves the metal efflux P_1_B-type ATPase CopA, which translocates Cu^+^ and Ag^+^ from the cytoplasm to the periplasm (4, 9). Therefore, the R. gelatinosus efflux-defective *copA* mutant was used to check the involvement of CopA in Ag^+^ efflux. Unlike CuSO_4_, which inhibits *copA* mutant growth (Fig. 2A), no difference in growth inhibition was observed between *copA* mutant and wild-type cells subjected to excess AgNO_3_. These data suggested that in contrast to Ag^+^ tolerance in E. coli, CopA is not involved in Ag^+^ tolerance in R. gelatinosus. Although the ZntA/CadA ATPase is known to translocate divalent cations, we also checked whether the Δ*cadA* mutant was sensitive to Ag+ (A. S. Steunou, A. Durand, M.L. Bourbon, M. Babot, S. Liotenberg, and S. Ouchane, submitted for publication). As for the *copA* mutant strain, no difference in growth was observed between wild-type and Δ*cadA* strains in the presence of AgNO_3_ (not shown). Cells were also spotted on solid medium supplemented with the same metals. Both *copA* and Δ*cadA* mutants showed growth inhibition on 500 µM CuSO_4_ and 500 µM CdCl_2_, respectively. However, none of the mutants exhibited an altered growth phenotype on 5 µM AgNO_3_ (Fig. 2B). We therefore concluded that the CopA and CadA ATPases were not involved in the AgNO_3_ response. To further support this conclusion, we analyzed by Western blot the expression of CopA and CopI in response to metal shock in a strain expressing a His-tagged version of CopA (CopA-H_6_) (Fig. 2C). Cells were grown under photosynthetic condition and shocked with CuSO_4_ and CdCl_2_, known to induce the expression of the Cu^+^ efflux system (Steunou et al., submitted for publication), or with AgNO_3_. Untreated cells showed a basal level of CopA and CopI expression because of the presence of 1.6 µM CuSO_4_ in the growth medium. As expected, addition of CuSO_4_ or CdCl_2_ to the growing cells led to significant increases in the amounts of CopA and CopI (Fig. 2C). In contrast, AgNO_3_ did not induce the expression of both proteins. Collectively, these results showed that the R. gelatinosus CopA efflux ATPase is not involved in AgNO_3_ stress response and detoxification, in contrast to that in E. coli.

10.1128/mBio.01535-18.1 FIG S1 Toxicity of CuSO_4_ and AgNO_3_ in high-density culture. Download FIG S1, PDF file, 0.1 MB. Copyright © 2018 Tambosi et al. 2018 Tambosi et al. This content is distributed under the terms of the Creative Commons Attribution 4.0 International license.

### Silver and copper excess specifically affected the LH2 complexes in the membrane.

**FIG 3:**
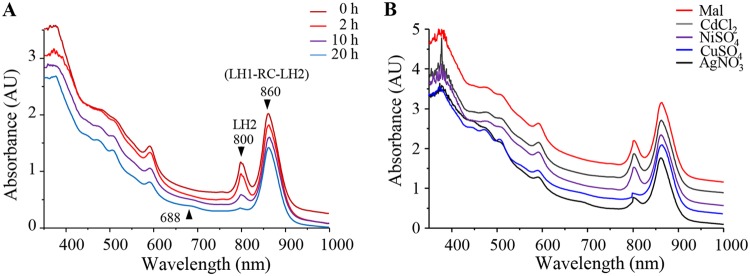
AgNO_3_ exposure impact on photosystem *in vivo*. (A) Spectral analyses of wild type (WT) cells grown overnight by photosynthesis and exposed or not to 1 mM AgNO_3_ after they reach an OD_680_ of 2. Spectra (350 to 1,000 nm) were recorded after 2- (light red line), 10- (purple line), or 20-h (blue line) exposure on a double-beam Cary 500 spectrophotometer. (B) Spectral analyses of the WT cells exposed or not to 1 mM CdCl_2_ (gray line), NiSO_4_ (purple line), CuSO_4_ (blue line), or AgNO_3_ (black line) after they reached an OD_680_ of 2. Spectra were recorded after 20 h of exposure. Mal (dark red line), untreated cells grown in malate medium.

To assess the effects of Ag^+^ ions on photosynthesis, cells (optical density at 680 nm [OD_680_] = 2) grown overnight under photosynthetic condition were treated with 1 mM AgNO_3_ and grown further for 2, 10, or 20 h. The bacteriochlorophyll *a* absorbance in the photosynthetic complexes was measured to monitor changes in response to excess AgNO_3_ in the cell in the reaction center and in light-harvesting antenna LH1 complexes and LH2 complexes. The effect of Ag^+^ on the photosynthetic (PS) complex spectra are presented in Fig. 3A. The B860 (RC-LH1-LH2) and B800 (LH2) wavelength band intensity variations, depending on the length of AgNO_3_ exposure, are represented. Ag^+^ induced no apparent effect on the 860-nm band. However, a time exposure-dependent decrease of the B800 band intensity was observed (Fig. 3A). This suggested that the LH2 antennae were affected by AgNO_3_ exposure. We also question whether this effect was specific to AgNO_3_. For that purpose, cells were also subjected to metal excess stress as described above, but with different metal cations (Fig. 3B). Interestingly, only CuSO_4_ caused the same effect as AgNO_3_ on the LH2 complexes. Exposure to CdCl_2_ or NiSO_4_ did not affect the photosynthetic complexes. These data demonstrated that AgNO_3_ and CuSO_4_ extended exposure affected the LH2 in the photosynthetic membranes.

### Silver and copper specifically induced the loss of the 800-nm absorbing bacteriochlorophyll *a* in LH2.

**FIG 4:**
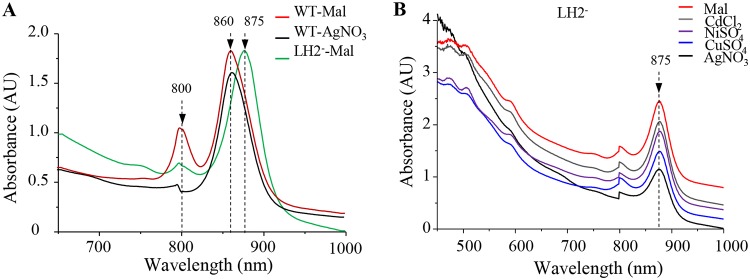
Effect of AgNO_3_ on LH2 complexes *in vivo*. (A) Absorption spectra of wild-type (WT) cells grown overnight by photosynthesis untreated (Mal, red line) or exposed to 1 mM AgNO_3_ (black line) in comparison with LH2-deficient mutant (LH2^−^) cells grown overnight by photosynthesis in malate medium (green line). (B) Spectral analyses of the LH2-deficient mutant cells untreated (Mal, red line) or exposed to 1 mM CdCl_2_ (gray line), NiSO_3_ (purple line), CuSO_4_ (blue line), or AgNO_3_ (black line) after they reach an OD_680_ of 2. Spectra were recorded after 20 h of exposure.

The LH2 antenna (B800 and B850) complexes are spectrally characterized by the 800- and 850-nm absorption bands that arise from the near-infrared (Qy) transitions of the bacteriochlorophyll *a*. The loss of the B800 band suggested the loss of the LH2 in the membrane. However, the spectra presented in Fig. 3 also suggested that the LH2 B850 bacteriochlorophyll was not affected, since no shift was observed in the 860-nm band that encompasses the RC (870-nm), the LH1 (875-nm), and the LH2 (850-nm) bands. To confirm this assumption, we compared the spectra of the untreated or AgNO_3_-shocked wild-type cells to the spectrum of the *pucBA* LH2-deficient mutant (28) that only assembles the RC-LH1 core (Fig. 4A). Deletion of the LH2 genes resulted in a significant decrease of the 800-nm band and a substantial red shift of 15 nm (from 860 to 875 nm) of the 860-nm band (28). The resulting peak at 875 nm corresponds to the RC-LH1 core absorption bands. In sharp contrast with the LH2-deficient mutant, the AgNO_3_-shocked wild-type cell spectrum showed the decrease of the 800-nm band and no changes in the 860-nm-absorbing bacteriochlorophyll molecules arising from the RC-LH1 core and a modified (B800-free) LH2 (Fig. 4A). Similar impact on LH2 complexes was observed when cells were subjected to CuSO_4_ treatment (Fig. S2). These spectra showed that the B800 molecules can selectively be extracted or released from the LH2 complexes in the presence of metals without disrupting the interaction of the LH2 polypeptides with the B850 molecules.

10.1128/mBio.01535-18.2 FIG S2 Effect of CuSO_4_ on LH2 complexes. Download FIG S2, PDF file, 0.1 MB. Copyright © 2018 Tambosi et al. 2018 Tambosi et al. This content is distributed under the terms of the Creative Commons Attribution 4.0 International license.

The effect of metals on the RC-LH1 core was also assessed using the *pucBA* LH2-deficient mutant. Spectra of the exposed cells showed that AgNO_3_ and CuSO_4_ slightly affected the amount of the RC-LH1 (Fig. 4B). Moreover, total protein lysates from all untreated or treated samples were also loaded onto SDS-PAGE. The Coomassie blue staining showed that all wild-type samples have comparable amounts of LH2 subunits, indicating that the release of B800 molecules did not affect the LH2 protein stability (Fig. S2). Altogether, these data indicated that AgNO_3_- and CuSO_4_-induced alterations in the structure of the LH2 complexes, targeting the B800 molecules of the complex. However, this specific and rather limited effect on B800 and the LH2 could not explain the drastic growth inhibition by Ag^+^, suggesting that Ag+ affects other crucial components or complexes of the cell.

### Metal-specific impact on the 800-nm band attested by the release of bacteriochlorophyll and shift in carotenoid absorbance.

**FIG 5:**
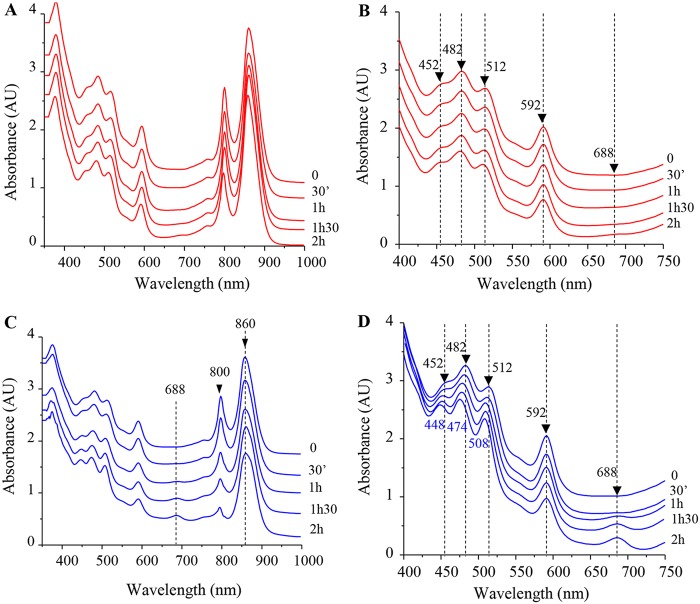
Effect of CuSO_4_ on LH2 complexes of isolated membranes. (A) Spectra (350 to 1,000 nm) of untreated membranes were recorded every 30 min. (B) Enlargement of the 400- to 750-nm spectrum absorbance region of untreated membranes. (C) Enriched membrane fractions were mixed with CuSO_4_ at 2 mM final concentration; 350- to 1,000-nm spectra were then recorded every 30 min. (D) Enlargement of the 400- to 750-nm spectrum absorbance region, highlighting the shift in the carotenoids bands and the increase in the 688-nm band in the CuSO_4_-treated membranes.

In the LH2 structure of Rhodopseudomonas acidophila, the 800-nm absorbing bacteriochlorophyll *a* molecules lie between the β-apoprotein helices, where phytyl moieties interact with the carotenoids. The structural data showed that at least one of the carotenoid molecules makes close Van der Waals contacts with the B800 pigment (25, 29). We therefore assumed that the release of B800 molecules following metal stress should also impact the B800-carotenoid interaction. To verify this assumption, enriched membranes from wild-type cells were incubated in phosphate buffer supplemented or not with 2 mM CuSO_4_ or AgNO_3_. Spectra were then recorded every 30 min to monitor the effect of metals on photosynthetic complexes on isolated membranes (Fig. 5). For untreated membranes, no changes in the amount or in the spectral properties of the RC, LH1, and LH2 were observed (Fig. 5A and B). However, in the membranes subjected to CuSO_4_ treatment, a significant decrease in the intensity of the 800-nm band was observed in association with an absorption increase at 688 nm (Fig. 5C and D). This later absorption peak very likely arose from oxidized Bchl in solution, as previously reported (30). Furthermore, with extended exposure to CuSO_4_, a shift was also observed in the carotenoid absorption region. Indeed, untreated proteins exhibited three peaks at 452, 482, and 512 nm (Fig. 5B), while CuSO_4_ treatment resulted in a shift of the carotenoid absorbance to 448, 474, and 508 nm (Fig. 5D). Similar effects were obtained in the presence of AgNO_3_ treatment (Fig. S3). Thus, we concluded that in the presence of CuSO_4_ or AgNO_3_, changes in the LH2 absorption spectrum are related to the disruption of the interaction of B800 molecules with LH2, which causes the release of the B800 molecules and subsequently impacts the spectral properties of the carotenoids within the B850 LH2.

10.1128/mBio.01535-18.3 FIG S3 Effect of AgNO_3_ treatment on LH2 complexes in purified membranes. Download FIG S3, PDF file, 0.1 MB. Copyright © 2018 Tambosi et al. 2018 Tambosi et al. This content is distributed under the terms of the Creative Commons Attribution 4.0 International license.

### Silver damages the cytochrome *c* oxidase and the succinate dehydrogenase in the respiratory chain.

**FIG 6:**
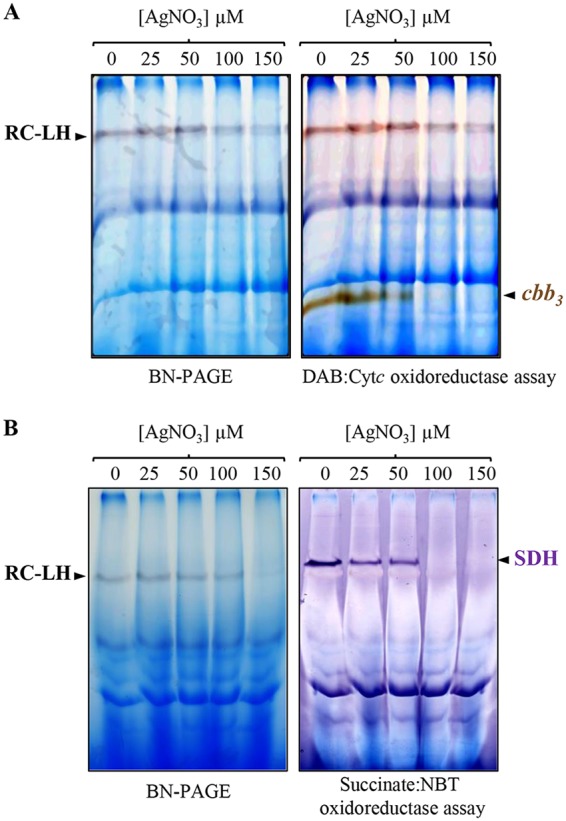
AgNO_3_ effect on respiratory complexes *in vivo*. The wild-type (WT) cells were grown under microaerobic respiratory condition and shocked for 1 h with increasing concentration of AgNO_3_. DDM-solubilized membrane proteins were separated on a 5% to 12% BN-PAGE. (A) *cbb*_3_ cytochrome *c* oxidase DAB:Cyt*c* in-gel activity assay. (B) Succinate dehydrogenase (SDH) in-gel activity assay.

Previous studies in 1974 and 2005 established that Ag^+^ ions inhibit the respiratory chain of E. coli (31, 32). In eukaryotes, Ag^+^ ions can induce mitochondrial dysfunction, partly by inhibiting respiration (33, 34). However, the complexes targeted by Ag^+^ ions were not yet identified. To check the effect of AgNO_3_ on two respiratory complexes (succinate dehydrogenase and *cbb*_3_ cytochrome *c* oxidase) from R. gelatinosus, exponentially growing wild-type cells under respiratory conditions were subjected to increasing concentration of AgNO_3_ (25 to 150 µM) and grown for another hour. To examine the effect of AgNO_3_ on the *cbb*_3_ oxidase, membrane proteins were solubilized and cytochrome *c* oxidase activity was assayed on blue native PAGE (BN-PAGE) (35). As shown in Fig. 6A, comparable diaminobenzidine (DAB)-positive bands corresponding to the *cbb*_3_ oxidase were revealed in the solubilized membrane proteins from untreated and 25 µM AgNO_3_-stressed cells. Decreased activity was detected in the 50 µM AgNO_3_-stressed cells. However, no active *cbb*_3_ oxidase was detected on membrane fractions isolated from 100 and 150 µM AgNO_3_-stressed cells (Fig. 6A). We should note that there was a slight effect on the amount of RC-LH with 100 and 150 µM AgNO_3_. This could be the consequence of membrane protein solubilization; indeed, the loss of B800 may destabilize LH2 in the presence of detergent. Nonetheless, the other blue-stained complexes on BN-PAGE did not seem to be affected (Fig. 6A and B), suggesting that AgNO_3_ targets only some complexes, including the *cbb*_3_ cytochrome *c* oxidase and the RC-LH. Similarly, succinate:nitroblue tetrazolium (NBT) *in gel* assay revealed an active band, likely corresponding to succinate dehydrogenase (SDH). Treatment with increasing concentration of AgNO_3_ resulted in a partial or full inhibition of this activity (Fig. 6B) suggesting that AgNO_3_ also affected succinate dehydrogenase in exposed cells.

**FIG 7:**
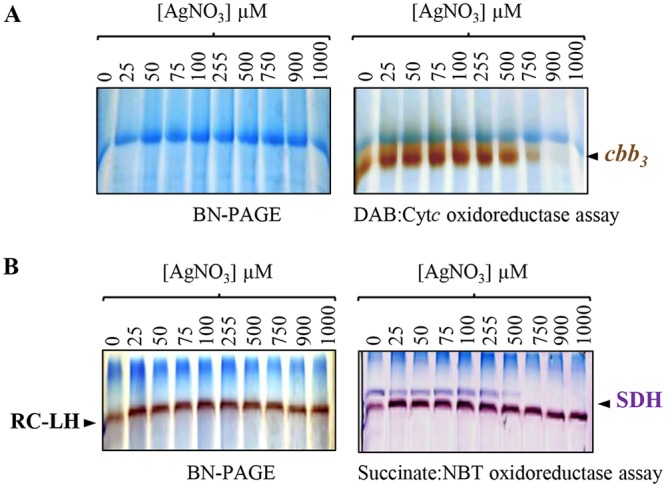
Effect of AgNO_3_ treatment on respiratory complexes in membrane-enriched fractions. These fractions were mixed with increasing concentration of AgNO_3_ for 1 h. DDM-solubilized membrane proteins were then separated on a 5% to 12% gradient BN-PAGE. (A) *cbb*_3_ cytochrome *c* oxidase in-gel activity assay. (B) Succinate dehydrogenase (SDH) in-gel activity assay.

As for photosynthetic complexes, we analyzed the *in vitro* effect of AgNO_3_ on respiratory complexes in isolated membrane protein fractions (Fig. 7). For that purpose, membranes from wild-type cells were incubated in buffer supplemented or not with increasing concentration of AgNO_3_ (from 25 to 1,000 µM). Similarly to the *in vivo* data, incubation of membrane proteins with increasing concentration of AgNO_3_ led to a decrease in cytochrome *c* oxidase *cbb*_3_ (Fig. 7A) and succinate dehydrogenase activities (Fig. 7B). Altogether, these data suggested that AgNO_3_ could inhibit respiration by directly damaging the respiratory complexes, including cytochrome *c* oxidase and succinate dehydrogenase.

### Silver damages respiratory complexes in Escherichia coli but not in Bacillus subtilis.

**FIG 8:**
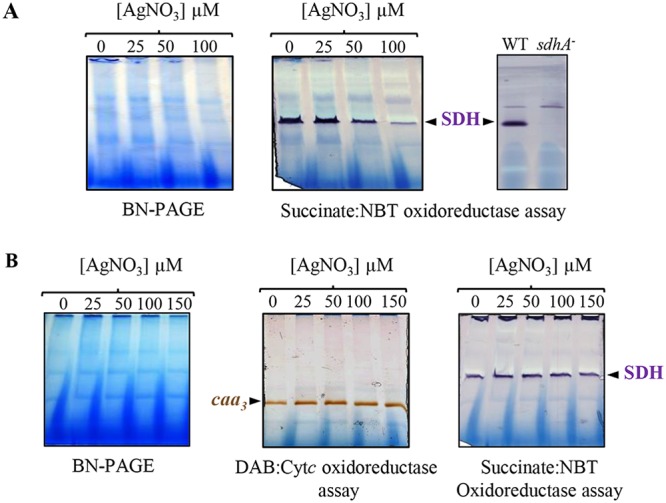
AgNO_3_ effect on respiratory complexes in E. coli and B. subtilis cells. Strains were grown under aerobic respiratory condition and shocked for 1h with increasing concentration of AgNO_3_. DDM-solubilized membrane proteins were separated on a 5% to 12% gradient BN-PAGE. (A) Succinate dehydrogenase (SDH) in-gel activity assay of E. coli membrane fractions. Membrane fraction from the SDH-deficient mutant (*sdhA^−^*) was used as a control. (B) *caa*_3_ cytochrome *c* oxidase and succinate dehydrogenase (SDH) in-gel activity assays of B. subtilis membranes.

The findings above prompted us to test the activity of respiratory complexes in other bacterial species after AgNO_3_ treatment. To this aim, E. coli and Bacillus subtilis cells grown to exponential phase were subjected to increasing concentration of AgNO_3_ (25 to 150 µM) and grown for another hour. Membranes proteins were isolated, and activity assays for respiratory complexes were performed by BN-PAGE. As E. coli cells do not express any cytochrome *c* oxidase, we only assayed the activity of succinate dehydrogenase. We detected changes in the activity of this complex when cells were subjected to AgNO_3_ stress, as the SDH activity decreased with increasing concentration of AgNO_3_ (Fig. 8A). To ascertain that the detected band corresponds to the SDH, the succinate-NBT in-gel assay was also performed with membrane proteins isolated from the *sdhA* deletion mutant (36) (Fig. 8A). These results confirmed that AgNO_3_ can affect respiration in E. coli and provide evidences that the SDH complex is a target of AgNO_3_. In B. subtilis, however, AgNO_3_ treatment did not affect the activity of the cytochrome *c* oxidase *caa*_3_, nor the activity of the SDH (Fig. 8B).

## DISCUSSION

**FIG 9:**
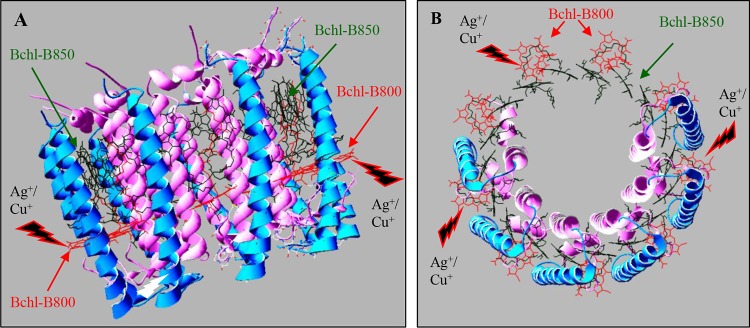
Ag^+^ and Cu^+^ specifically target the B800 within the LH2. Structure of the LH2 and arrangement of pigments within the complex from Rhodopseudomonas acidophila (1KZU.pdb) (25). The figure was generated using the Protein Data Bank (Swiss-PdbViewer). View parallel to the plane of the membrane (A) and from the top of the complex (B) showing the exposed B800 molecules (in red) located between the β subunit helices, which form the outer ring (cyan). The B850 molecules (green) are buried between the concentric rings formed by the helices of the α subunits (pink) and the outer β subunit helix ring. For better viewing, carotenoids and helices of α and β subunits were hidden.

The use and spread of metal ions or nanoparticles represent a serious threat to the environment and to all living organisms because of the acute toxicity of these ions. Silver and copper ions have been used for their antimicrobial activities for several years. Nowadays, Ag^+^ nanoparticles are one of the most widely used nanoparticles in many industrial and health applications (37). The antimicrobial effect of Ag^+^ nanoparticles is in part related to the released Ag^+^ ions and their ability to interact with bacterial membranes (37–39). It is therefore important to characterize the toxicity of Ag^+^ ions and to identify cellular targets of this metal. Previous studies reported that the acute toxicity of Ag^+^ lies in its ability to interact with membranes but also in its ability to affect iron sulfur proteins (13). To identify targets of Ag^+^, we compared the impact of different metal ions on the stability and activity of membrane complexes in the purple photosynthetic bacterium R. gelatinosus. AgNO_3_ was found to be more toxic than the other ions used, including CuSO_4_ and CdCl_2_. This may be related to the absence of an efficient efflux system to detoxify Ag^+^ and/or to its bioactivity and ability to damage molecules. Indeed, the Cop system involved in detoxification of Ag^+^ in other bacteria (9–11) is not induced by AgNO_3_ in R. gelatinosus, which may increase the susceptibility of the bacterium to AgNO_3_. In E. coli, although the Cop efflux is effective in expelling Ag^+^ ions outside the cells, AgNO_3_ remains very toxic and targets different cellular components. In Chlamydomonas reinhardtii (39, 40) and Arabidopsis thaliana (41), Ag^+^ and Cu^+^ exposures were both found to significantly inhibit growth and to induce decreases in photosynthesis and chlorophyll content. Here, we found that both metals target the bacterial LH2. Both Ag^+^ and Cu^2+^ specifically target the B800 molecules but not the B850 ones (Fig. 9). Similar results were reported when LH2 complexes from R. sphaeroides and R. acidophila were subjected to high atmospheric pressure (30). This could be related to the structure of this complex and the position of the chlorophyll molecules in the complex. In fact, the B850 bacteriochlorophylls with the carotenoid molecules are buried between the concentric rings formed by the α and β subunit outer rings (25) and are therefore well shielded from the external buffer. In contrast, the B800 molecules are located between the outer rings formed by helices of the β subunits and are parallel to the lipid surface near the cytosolic side (Fig. 9). This positions the B800 molecules in contact with the solvent, where they would be more exposed than the B850 molecules, in agreement with water molecules being found close to the B800 molecules in the R. acidophila LH2 crystal structure (25, 29). The effect on B800 did not modify the complex stability, since the B850 molecules were not affected. Nevertheless, the resulting complex would be inefficient for light energy capture and photon transfer to the photochemical reaction center. Indeed, the LH2 light energy is transferred from B800 to B850. Energy transfer then occurs between B850 and B875 molecules in the light-harvesting complex LH1 to the RC (42). Thus, we assume that Ag^+^ or Cu^+^ exposure will result in reduced excitation transfer to the B870 in the RC and decreased photosynthesis yield. Only a slight effect of Ag^+^ and Cu^+^ was observed on the LH1-RC in the LH2-deficient strain. In the LH1-RC structures from R. palustris and T. tepidum (26, 27), the bacteriochlorophyll molecules in LH1 complexes are found sandwiched between the concentric ring formed by the α subunits and the external ring formed by the β helices, like the B850 molecules in the LH2 structure. These Bchl molecules and the RC-Bchls could therefore be shielded from the external buffer and therefore from damages that may be caused by the presence of metal ions. The mechanism by which Ag^+^ or Cu^+^ release B800 from the LH2 remains to be studied. Nonetheless, previous studies have shown that Mg^2+^ in chlorophylls could be substituted, both *in vitro* and *in vivo*, by heavy metal ions (20, 43, 44). It was shown that *in vivo* substitution of the Mg^2+^ atom of chlorophyll by heavy metals, including Cu^+^, Cd^2+^, or Pb^2+^, is a major damage mechanism in stressed plants. Indeed, substitution of Mg^2+^ affects the LHCII and the photosystem PSII, thereby causing a decrease in photosynthesis (20). Likewise, it was shown that Ni^2+^, Cu^2+^, and Zn^2+^ induced a destabilization of heme binding to *b*-type hemoproteins and led to the release of heme from myoglobin, ferricytochrome *b_5_*, indoleamine-dioxygenase, hemopexin, and cytochrome P450 (45, 46). Formation of a bioconjugate of human hemoglobin with Ag^+^ ions was also reported (47, 48). Finally, both Ag^+^ and Cu^+^ can displace metal- or damage-exposed 4Fe-4S clusters in proteins (13). By theses means, such metal ions can inhibit the activity of hemoproteins and metaloproteins in the membrane and the cytosol. AgNO_3_ impacts on the activity of cytochrome *c* oxidase and succinate dehydrogenase in R. gelatinosus, as well as on the succinate dehydrogenase in E. coli, were demonstrated in this study. This could arise from the disruption of the interaction between the cofactors (heme or 4Fe-4S) and the proteins. Nevertheless, we should note that the effects of Ag^+^ on complexes reported in this study were obtained with high concentration of AgNO_3_. No effect was shown in the Gram-positive bacterium B. subtilis. This may be related to the difference in the cell wall structure between Gram-positive and Gram-negative bacteria. The much thicker peptidoglycan layers in Gram-positive bacteria are crucial in protecting the cell from environmental stress, including that of external metal ions (49). In agreement with this, Staphylococcus aureus is less sensitive to AgNO_3_ than E. coli, as AgNO_3_ treatment was shown to strongly affect the membrane integrity of E. coli but not that of S. aureus (50–52). In a recent study, AgNO_3_ was shown to affect the activity of the cytochrome *c* oxidase in B. subtilis (53). However, AgNO_3_ interfered with the biogenesis process of the oxidase by displacing Cu^2+^ from the Sco assembly protein (53). In our study, we checked the impact of AgNO_3_ on already-assembled complexes. Nevertheless, prolonged exposure to metal should be further studied to better characterize the response to Ag^+^ stress in B. subtilis. Beside the direct effect of Cu^2+^ and Ag^+^ on these membrane complexes, heme or 4Fe-4S cluster degradation is expected to release iron, which may magnify the toxicity of metals, as excess free iron gives rise to hydroxyl radicals and induces oxidative stress. Most toxicity studies of Ag nanoparticles in bacteria and eukaryotes shed light on oxidative stress response. For a full characterization of nanoparticle toxicity mechanisms, future experiments should address the issue of interaction between membrane complexes involved in cellular bioenergetics and Ag nanoparticles.

## MATERIALS AND METHODS

### Bacterial strains and growth.

E. coli and B. subtilis cells were grown aerobically (500-ml flasks containing 50 ml medium) at 37°C in LB medium. R. gelatinosus cells were grown at 30°C, in the dark microaerobically (low oxygenation in 50-ml flasks containing 50 ml medium) or in light by photosynthesis (filled tubes with residual oxygen in the medium) in malate growth medium (54). The antibiotics kanamycin (Kan) and trimethoprim (Tmp) were used at a final concentration of 50 µg/ml.

Growth inhibition curves were monitored at OD_680_, with measurements taken every 15 min for 24 h, using an Infinite M200 luminometer (Tecan, Mannerdorf, Switzerland) for aerobic condition. For photosynthesis conditions, strains were grown as described above, and OD was measured after 24 h using the Tecan luminometer.

### Membrane protein preparation.

Cells were disrupted by sonication in 0.1 M sodium phosphate buffer (pH 7.4) containing 1 mM phenylmethylsulfonyl fluoride. Unbroken cells were removed by a low-speed centrifugation step (25,000 × *g*, 30 min, 4°C), and supernatants were subjected to ultracentrifugation (200,000 × *g*, 90 min, 4°C) to collect the membrane fraction. Membrane fractions were then resuspended in the same buffer. Membrane protein concentration was estimated using the bicinchoninic acid assay (Sigma), with bovine serum albumin as the standard. For membrane protein metal treatment, required concentrations of metal solution were mixed with 50 mg/ml membrane proteins at room temperature. Spectra were recorded every 30 min.

### Spectrophotometric measurements.

Absorption spectroscopy was performed with a Cary 500 spectrophotometer. For spectra on whole cells, cells were resuspended in a 60% (wt/vol) sucrose solution. Membrane fractions were in 0.1 M sodium phosphate buffer (pH 7.4).

### Blue native gel electrophoresis and in-gel assays.

To assay *cbb*_3_ and succinate dehydrogenase activities, R. gelatinosus wild-type cells were grown microaerobically. For E. coli and B. subtilis, cells were grown aerobically. Membranes were prepared as previously described. Blue native polyacrylamide gel electrophoresis (BN-PAGE) and in-gel Cox activity assays (DAB:Cyt*c* staining) were performed as described in (35), and succinate dehydrogenase activity was assayed using succinate and NBT (nitroblue tetrazolium), as described in reference (55).

### Western blot analysis and HisProbe-HRP detection.

Equal amounts of cells (OD_680_ = 1) were disrupted in SDS loading buffer, and proteins were then separated on a 15% SDS-PAGE and further transferred to a Hybond ECL polyvinylidene difluoride (PVDF) membrane (GE Healthcare). Membranes were then probed with the HisProbe-horseradish peroxidase (HRP) (Pierce), according to the manufacturer’s instructions, and positive bands were detected using a chemiluminescent HRP substrate, according to the method of Haan and Behrmann (56). Image capture was performed with a ChemiDoc camera system (Bio-Rad).
