# Supplementary material for: Silver and Copper Acute Effects on Membrane Proteins and Impact on Photosynthetic and Respiratory Complexes in Bacteria
Source: mBio. 2018 Nov 20;9(6):e01535-18. doi: 10.1128/mBio.01535-18 (PMC6247083; doi:10.1128/mBio.01535-18)
Supplement: FIG S1 [file mbo006184167sf1.pdf]

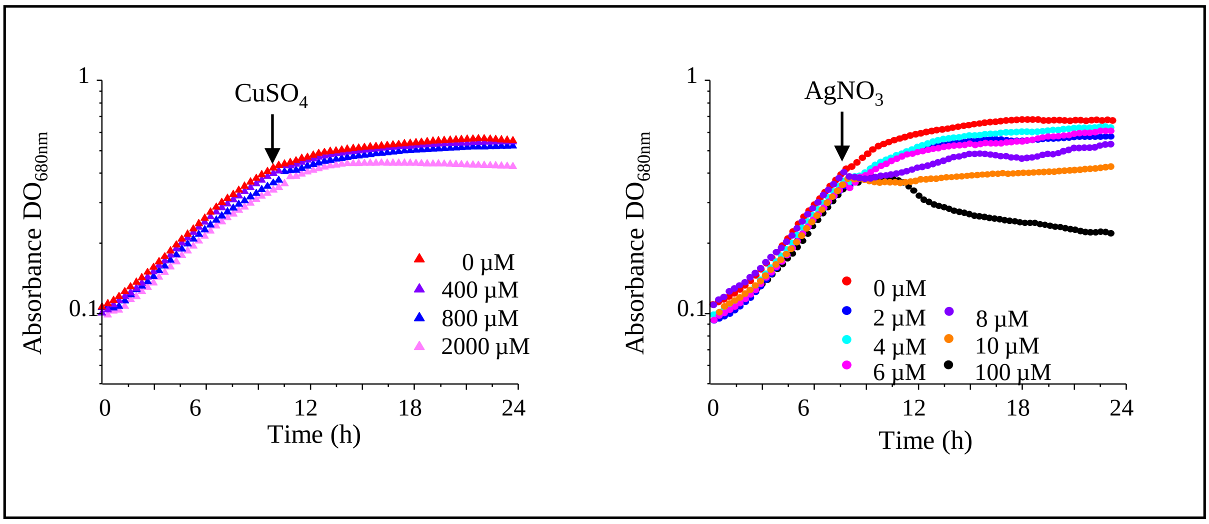

Fig. S1: Toxicity of $CuSO_4$ and $AgNO_3$ in high cell density culture. Cells were grown in micro-aerobisis on microplates in the Tecan Infinite M200 luminometer. Indicated concentrations of $CuSO_4$ or $AgNO_3$ were added to the growth medium after cells reached OD680nm=0.4 (arrow).
