# Supplementary material for: Silver and Copper Acute Effects on Membrane Proteins and Impact on Photosynthetic and Respiratory Complexes in Bacteria
Source: mBio. 2018 Nov 20;9(6):e01535-18. doi: 10.1128/mBio.01535-18 (PMC6247083; doi:10.1128/mBio.01535-18)
Supplement: FIG S2 [file mbo006184167sf2.pdf]

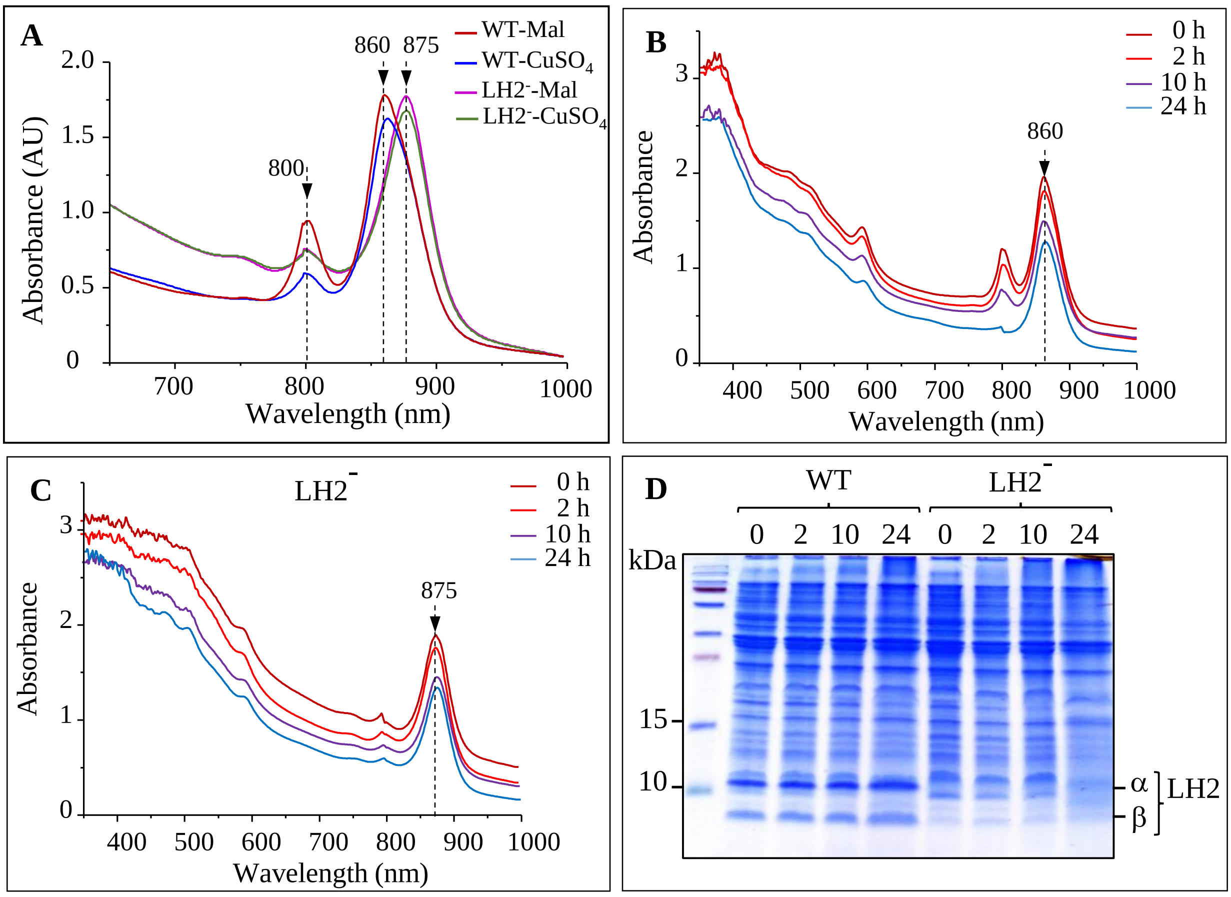

Fig. S2: Effect of $CuSO_4$ on LH2 complex. **A-** Absorption spectra of WT and LH2 deficient mutant cells grown overnight under photosynthetic condition and untreated (-Mal, red and magenta lines for the WT and LH2$^-$ strains respectively) or exposed to 1 mM $CuSO_4$ (blue and green lines for the WT and LH2$^-$ strains respectively**). B-** Absorption spectra of WT cells grown overnight under photosynthetic condition and exposed to 1 mM $CuSO_4$. The 350-1000 nm spectra were recorded after 2, 10 and 24 h. **C-** Spectral analyses of the LH2 deficient mutant cells exposed to 1 mM $CuSO_4$. Spectra were recorded after 2, 10 and 24 h. **D-** Coomassie blue staining of total protein lysate from untreated or $CuSO_4$ treated cells.
