# Supplementary material for: Silver and Copper Acute Effects on Membrane Proteins and Impact on Photosynthetic and Respiratory Complexes in Bacteria
Source: mBio. 2018 Nov 20;9(6):e01535-18. doi: 10.1128/mBio.01535-18 (PMC6247083; doi:10.1128/mBio.01535-18)
Supplement: FIG S3 [file mbo006184167sf3.pdf]

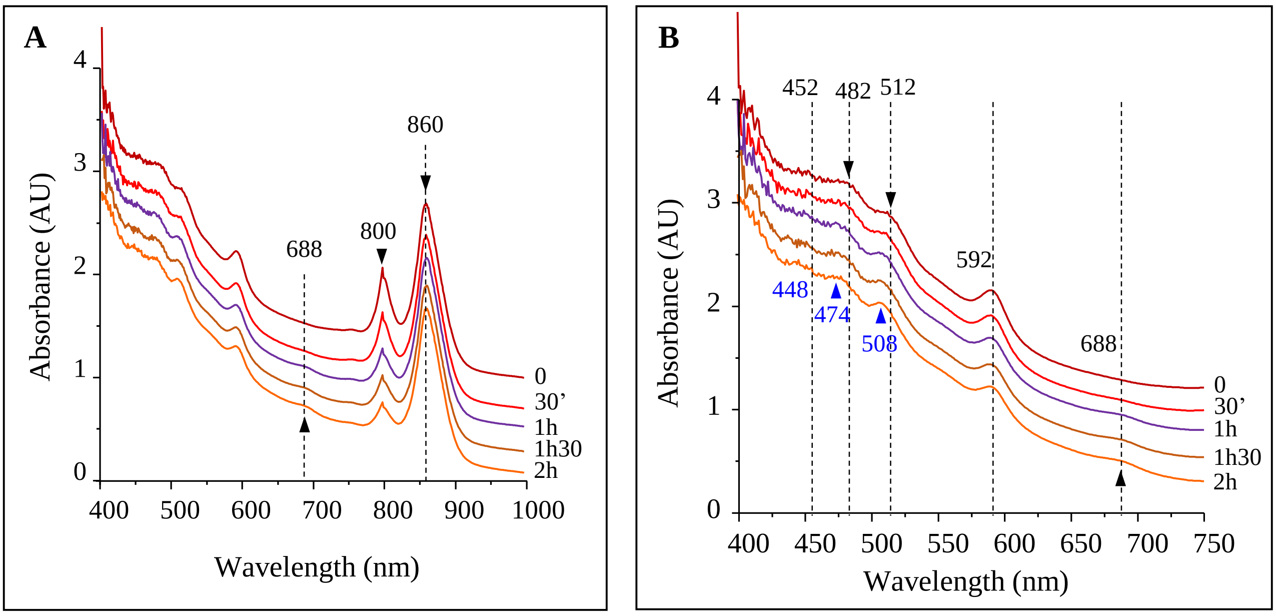

Fig. S3: Effect of $AgNO_3$ treatment on LH2 complexes in membrane-enriched fractions.
A- Membrane fractions were mixed with $AgNO_3$ at a final concentration of 2 mM and 350-1000 nm spectra were recorded every 30 min. **B-** Zoom in the 400 -750 nm spectrum absorbance region highlighting the shift in the carotenoid bands and the increase in the 688 nm band.
